# FluxRETAP: a REaction TArget Prioritization genome-scale modeling technique for selecting genetic targets

**DOI:** 10.1093/bioinformatics/btaf471

**Published:** 2025-08-23

**Authors:** Jeffrey J Czajka, Joonhoon Kim, Yinjie J Tang, Kyle R Pomraning, Aindrila Mukhopadhyay, Hector Garcia Martin

**Affiliations:** Energy and Environment Directorate, Pacific Northwest National Laboratory, Richland, WA, 99354, United States; US Department of Energy Agile BioFoundry, Emeryville, CA, 94608, United States; Energy and Environment Directorate, Pacific Northwest National Laboratory, Richland, WA, 99354, United States; US Department of Energy Agile BioFoundry, Emeryville, CA, 94608, United States; US Department of Energy Joint BioEnergy Institute, Emeryville, CA, 94608, United States; Department of Energy, Environmental and Chemical Engineering, Washington University, St. Louis, MO, 63130, United States; Energy and Environment Directorate, Pacific Northwest National Laboratory, Richland, WA, 99354, United States; US Department of Energy Agile BioFoundry, Emeryville, CA, 94608, United States; US Department of Energy Joint BioEnergy Institute, Emeryville, CA, 94608, United States; Biological Systems and Engineering Division, Lawrence Berkeley National Laboratory, Berkeley, CA, 94720, United States; Environmental Genomics and Systems Biology Division, Lawrence Berkeley National Laboratory, Berkeley, CA, 94720, United States; US Department of Energy Agile BioFoundry, Emeryville, CA, 94608, United States; US Department of Energy Joint BioEnergy Institute, Emeryville, CA, 94608, United States; Biological Systems and Engineering Division, Lawrence Berkeley National Laboratory, Berkeley, CA, 94720, United States; Environmental Genomics and Systems Biology Division, Lawrence Berkeley National Laboratory, Berkeley, CA, 94720, United States

## Abstract

**Motivation:**

Metabolic engineering is rapidly evolving as a result of new advances in synthetic biology tools and automation platforms that enable high throughput strain construction, as well as the development of machine learning tools (ML) for biology. However, selecting genetic engineering targets that effectively guide the metabolic engineering process is still challenging. ML can provide predictive power for synthetic biology, but current technical limitations prevent the independent use of ML approaches without previous biological knowledge.

**Results:**

Here, we present FluxRETAP, a simple and computationally inexpensive method that leverages the prior mechanistic knowledge embedded in genome-scale models for suggesting targets for genetic overexpression, downregulation or deletion, with the final goal of increasing the production of a desired metabolite. This method can provide a list of desirable engineering targets that can be combined with current ML pipelines. FluxRETAP captured 100% of reaction targets experimentally verified to improve *Escherichia coli* isoprenol production, 50% of targets that experimentally improved taxadiene production in *E. coli* and ∼60% of genetic targets from a verified minimal constrained cut-set in Pseudomonas putida, while providing additional high priority targets that could be tested. Overall, FluxRETAP is an efficient algorithm for identifying a prioritized list of testable genetic and reaction targets.

**Availability and implementation:**

FluxRETAP is implemented in python and released under the creative commons license. The implementation and code are freely available at: https://github.com/JBEI/FluxRETAP.

## 1 Introduction

Advances and parallelization in gene editing technologies are facilitating the rapid construction and testing of strains, propelling innovations in synthetic biology. Computational tools for strain design have become important components of the design-build-test-learn (DBTL) cycles that help select valuable gene targets aimed at enhancing the production of valuable chemicals (Nielsen and Keasling 2016, Carbonell *et al.* 2018, HamediRad *et al.* 2019, Banerjee *et al.* 2020, 2024, Zhang *et al.* 2020, Keasling *et al.* 2021). Genome-scale models (GSMs) are one such computational tool that encodes biological knowledge as a mathematical representation of metabolic pathways within a cell system. These mathematical representations offer a comprehensive way to link genes to reactions while providing a framework suitable to optimization methods. Mechanistic modeling approaches like COnstraint-Based Reconstruction and Analysis (COBRA) are then widely used to leverage GSMs to identify genetic targets for initial DBTL cycles (Burgard *et al.* 2003, Trinh *et al.* 2009, Chowdhury *et al.* 2015, Heirendt *et al.* 2019). The most commonly used approach is called flux balance analysis (FBA), where an objective function is optimized and results in a predicted flux distribution. However, due to the wide search space and inherent limitation of GSMs (e.g. no regulation, product toxicity or thermodynamic information), COBRA and FBA approaches can provide inaccurate flux distributions, especially for engineered strains. As such, several COBRA methods such as minimizations of metabolic adjustment (MOMA) (Segrè *et al.* 2002), regulatory on/off minimization of metabolic fluxes (ROOM) (Shlomi *et al.* 2005) and relative optimality of in metabolic networks (RELATCH) (Kim and Reed 2012) have been developed that use a reference flux state to improve accuracy of genetic selection of strains. Unfortunately, these algorithms can be computationally expensive to generate a large number of strains. Particularly, bi-level optimization methods like OptKnock (Burgard *et al.* 2003) or RobustKnock (Tepper and Shlomi 2010) can require long computational times for large models. More efficient algorithms have been developed that use a step-wise increase in product production and identify reactions that need to change to improve production [flux scanning based on enforced objective flux, FSEOF (Choi *et al.* 2010], [flux variability scanning based on enforced objective flux, FVSEOF (Park *et al.* 2012)]. These algorithms have been experimentally verified for several cases.

Here, we present Flux-REaction TArget Prioritization (FluxRETAP), a computationally inexpensive algorithm for target selection that generates hundreds of gene targets on the order of minutes. This algorithm has a similar initial implementation as the FSEOF/FVSEOF but has a different implementation for tracking and selecting target reactions (detailed in Section 2.1). FluxRETAP’s recommended targets are an ideal starting point to use with ML-guided active learning methods such as the Automated Recommendation Tool (Radivojević *et al.* 2020) or METIS (Pandi *et al.* 2022). Indeed, a precursor of FluxRETAP was used to identify targets in a machine learning pipeline which ultimately led to a 74% improvement in tryptophan titer (Zhang *et al.* 2020). A parallel study utilized FluxRETAP and resulted in significant improvements in isoprenol yields in *Pseudomonas putida*, with over 40% of returned targets resulting in improvements (Yunus *et al.* 2025).

## 2 Implementation

FluxRETAP has been implemented in Python and leverages functions and tools from the COBRA python (COBRApy) library (Heirendt *et al.* 2019). In particular, the algorithm utilizes the COBRApy input and output functions for handling GSMs and the flux variability analysis (FVA) optimization function to generate data for determining target reactions. Thus, FluxRETAP works with the standard COBRApy models and structures and is compatible with models incorporating additional constraints such as thermodynamic or enzyme expression limits, which could further improve target prediction accuracy. Users specify the COBRApy model structure and the final product, biomass, and carbon source. Optional parameters control the selectivity of the number of returned reactions. A detailed tutorial is provided via a Jupyter notebook in the github repository (https://github.com/JBEI/ FluxRETAP).

### 2.1 Workflow

**Figure 1.**
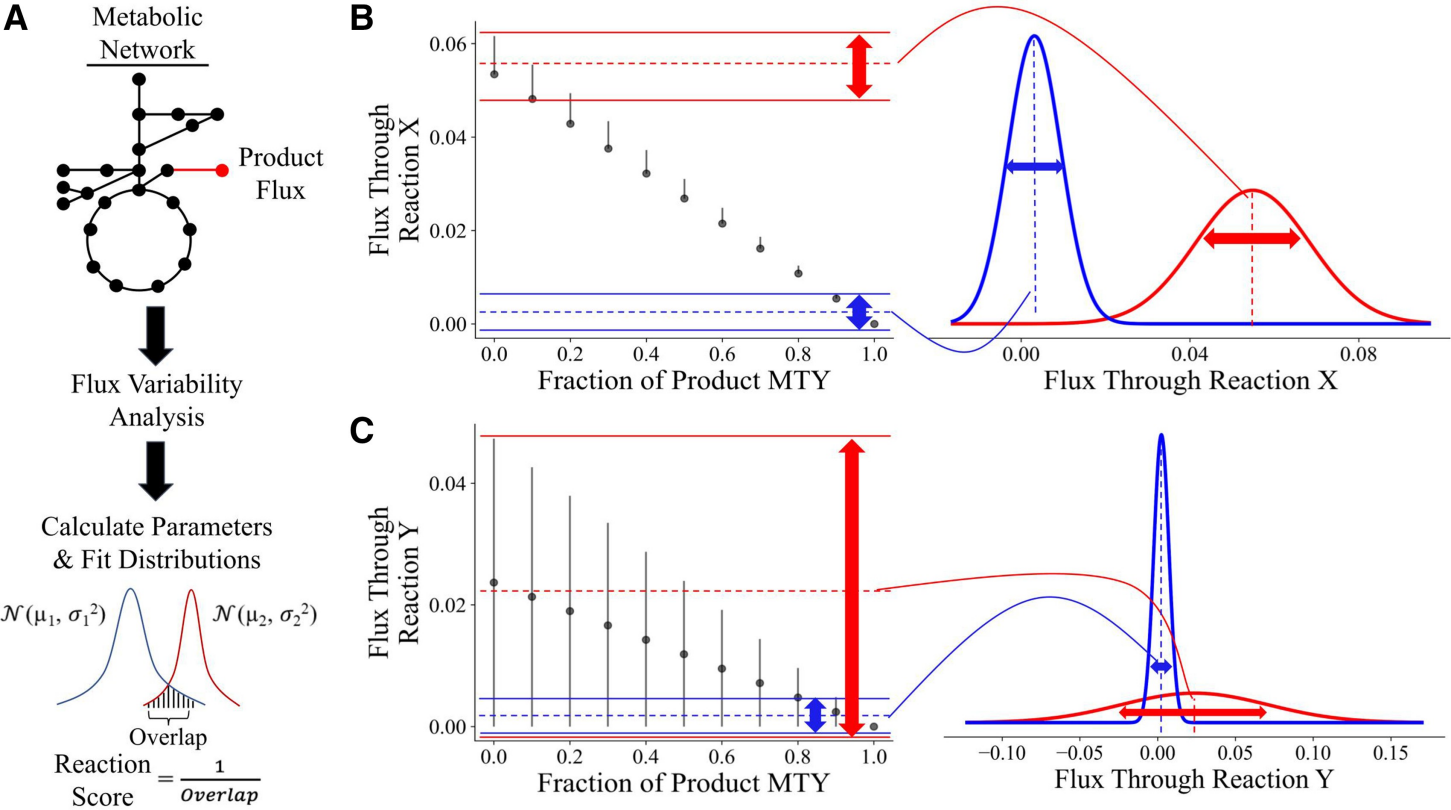
FluxRETAP algorithm explanation and examples. (A) Overview of FluxRETAP workflow: FluxRETAP performs FVA at several fractions of the MTY of production (e.g. at 10%, 20%, 30%, …, 100% of the maximum flux towards the final product), finding for each reaction the range of fluxes compatible with each fraction. FluxRETAP then calculates the overlap between initial (first two fractions, e.g. 10%, 20%) and final (last two fractions, e.g. 90%, 100%) flux distributions by fitting gaussian distributions to these flux ranges and finding their overlap. FluxRETAP uses as score the inverse of the overlap, because we are interested in the reactions for which there is the largest change between low production and high production (i.e. smallest overlap between initial and final flux distributions). We roughly approximate the mean and standard deviation (µ, σ) for the gaussian distributions as the average and the difference between largest and smallest fluxes, respectively. (B) When the gaussian fit to the initial fractions (red) has very little overlap with the gaussian fit to the final fractions (blue) we have a reaction of high interest (high score) since it needs to display very different values for low and high production. In this case the reaction must decrease to zero to enable high production. (C) When the gaussian fit to the initial fractions (red) has a large overlap with the gaussian fit to the final fractions (blue) we have a reaction of low interest (low score) since it could display the same value for low and high production.

FluxRETAP relies on a very simple idea: it tracks the flux span (flux range, or difference between the maximum and minimum feasible flux values) for each reaction as more flux is forced through the pathway synthesizing the desired product, a concept similar to those used in algorithms such as FSEOF (Choi *et al.* 2010) and FVSEOF (Park *et al.* 2012) (Fig. 1A). While FSEOF and FVSEOF consider flux trends, FluxRETAP quantifies the predicted overlap between low and high production states to rank reactions as desirable targets for genetic manipulations (Fig. 1B and C). Fluxes that need to be modified to produce high flux into the product are expected to have almost no overlap between the low producing and high producing cases. The initial step in FluxRETAP determines the maximum theoretical yield (MTY) of the final product in the GSM. Then, the algorithm steadily increases flux towards the final product from zero to the MTY and performs FVA for all the flux fractions. The maximum and minimum fluxes for the two lowest and highest fractions are used to fit gaussian distributions and determine their overlap. The mean for the gaussian (µ) is calculated as the average of the FVA range for the two initial (or last) fractions, while the standard deviation (σ) is the difference between the max and the min for those same fractions (Fig. 1B and C). The gaussians are used to determine overlap between the low and high producing states using the overlap index (Pastore and Calcagnì 2019). Reaction scores are reported as 1/overlap index, with a high score representing more confidence that a particular reaction is a desirable target because there is a clearer difference between low and high production of the final product. In practice, a high score means a reaction had to change in order for the GSM to reach a high production state. The score is used to rank the priority of the target.

The difference in flux mean values, as more material is forced through the final pathway (decreasing or increasing), determines what kind of genetic intervention is recommended (under or overexpression respectively). We do not expect every recommendation to be fruitful due to the limitations of GSM models and nonlinear metabolic effects. However, this is less of a problem in the current state of the technology, in which thousands of genetic edits are possible, than in the past, where a single gene knockout could take weeks to be generated (Garst *et al.* 2017, Bao *et al.* 2018, Hossain *et al.* 2020). By working with a large number of edits, machine learning algorithms can learn to avoid the recommendations that do not work (Culley *et al.* 2020, Radivojević *et al.* 2020, Zhang *et al.* 2020).

## 3 Application of FluxRETAP to experimental data

A precursor (proof-of-concept) version of FluxRETAP was used to identify targets in a machine learning pipeline, resulting in a 74% improvement in tryptophan titer (Zhang *et al.* 2020). To further assess the algorithm’s performance, we evaluated the ability of FluxRETAP to return computationally identified and experimentally verified reaction targets reported in the literature for *Escherichia coli* (Boghigian *et al.* 2012, Cotten and Reed 2013, Tian *et al.* 2019) and *P. putida* (Banerjee *et al.* 2020). FluxRETAP demonstrated a high-target capture rate with 100% of experimentally verified genes for an isoprenol producing *E. coli* strain (Tian *et al.* 2019) and 50% (two of four) tested targets in taxadiene producing *E. coli* (Boghigian *et al.* 2012) (Table 1). Four of the eight genes that improved isoprenol production were returned within the first 50 reaction targets, with six total returned within the first 100. In the taxadiene study, the two genes that were experimentally verified as improving production were returned as high priority targets (within the top four of targets to pursue). FluxRETAP was also able to capture ∼56% of experimentally verified genes for glutamine/indigoidine production in *P. putida* (Banerjee *et al.* 2020). Each simulation was completed in ∼30 s on a 2023 MacBook Pro with an Apple M2 Pro processor and 16 GB unified memory.

**Table 1.** Comparison of FluxRETAP, FSEOF, and FVSEOF results for three GSMs and targets.

Species/GSM	Product	Original method	Original method targets returned	Flux RETAP gene targets	FSEOF gene targets [overlap]	FVSEOF gene targets [overlap]	Reference
*E. coli*/iAF1260	Taxadiene	Constrained MOMA	12	56	70 [33]	9 [9]	Boghigian *et al.* (2012)
			Captured/validated targets	2/4	1/4	1/4	
*E. coli*/iJO1366	Isoprenol	Competing pathways	8	238	56 [38]	35 [33]	Tian *et al.* (2019)
			Captured/validated targets	8/8	4/8	1/8	
*P. putida/*iJN1463	Indigoidine	Constrained minimal cut set	16	386	528 [187]	384 [77]	Banerjee *et al.* (2020)
			Captured/validated targets	9/16	9/16	9/16	

The effects of varying FluxRETAP parameters (the minimal percent of biomass growth, the flux range difference cutoffs, reaction score cutoff values, etc.) on the number of returned reactions was examined using *E. coli*, *P. putida*, and *Saccharomyces cerevisiae* GSMs. We observed that the flux range difference and the threshold for returning reactions based on the score led to more stringent results, and less targets returned. Overall, these results indicated that FluxRETAP returns at a maximum ∼40%–50% of active (carrying non-zero flux) gene-associated reaction targets under the least stringent condition, whereas applying more selective search parameters limits targets to ∼2% of active reactions with an associated gene (see parmeterExploration.ipynb notebook).

## 4 Conclusion

FluxRETAP is a computationally inexpensive and intuitive algorithm that can generate genetic targets within 0.5–4 min for use in combination with active learning approaches guided by machine learning. The method leverages the biological knowledge encoded in genome-scale models through flux variability to identify reaction whose flux distributions are correlated with increasing production of the final product, allowing for the identification of genes targets that are either negatively correlated (down-regulation or knock-out targets) or positively correlated (overexpression targets). The algorithm captures a large portion of experimentally verified genes predicted by previous algorithms. The code has been implemented as a simple package in Python that allows users to easily generate targets in minutes, while also allowing users to apply more selective criteria in identifying reactions.

## Supplementary Material

btaf471_Supplementary_Data

## Data Availability

The data underlying this article are available in the article and in its online supplementary material. The code is available in the online repository at https://github.com/JBEI/FluxRETAP.
